# Insights into brain metastasis: Recent advances in circulating tumor cell research

**DOI:** 10.1002/cnr2.1239

**Published:** 2020-02-12

**Authors:** Remi Klotz, Min Yu

**Affiliations:** ^1^ Department of Stem Cell Biology and Regenerative Medicine Keck School of Medicine of the University of Southern California Los Angeles California; ^2^ USC Norris Comprehensive Cancer Center Keck School of Medicine of the University of Southern California Los Angeles California

**Keywords:** brain metastasis, circulating tumor cells, liquid biopsies

## Abstract

**Background:**

How tumor cells disseminate to brain and establish brain metastasis remains partly an unsolved problem. This devastating complication of many cancers is initiated by a rare subset of the circulating tumor cells (CTCs) shed into the blood stream. Thus, the profiling of the molecular properties in these brain metastasis‐initiating CTCs is essential to uncover the mechanisms underlying brain metastasis.

**Recent Findings:**

Important efforts to improve the enrichment and detection of CTCs enabled the detailed molecular and functional analysis of CTCs that drive brain metastasis. In this review, we highlight key findings on existing preclinical studies that provide insights toward a comprehensive picture of brain metastasis‐precursors in CTCs and the potential clinical implications.

**Conclusion:**

A deeper understanding of the brain metastasis precursors should help to stratify high‐risk patients and improve preventive therapeutic strategies. Although all these preclinical evidences have yet to be translated into patients, they provide considerable hope to benefit patients with brain metastases in the future.

## 1 INTRODUCTION

Brain metastasis refers to the development of an intracranial tumor following the seeding of tumor cells from primary tumor or established metastases outside of the central nervous system.^1^ Brain metastases can occur from any types of cancer; however the incidence rate varies by patient age, sex, source, and molecular subtype of the primary tumor.^2^, ^3^, ^4^, ^5^ According to several epidemiology studies, the most common primary tumors associated with high incidence of brain metastasis are lung (19%‐40%), melanoma (7%‐15%), and breast (5%‐20%).^2^, ^4^, ^6^ It is widely recognized that the true incidence is underestimated, as brain magnetic resonance imaging (MRI) screening is limited only to patients who present clinical symptoms and autopsy studies showed higher incidence of brain metastases in cancer patients.^1^, ^7^, ^8^ Brain metastasis has devastating prognosis and results in significant morbidity and mortality in patients, with median survival ranging from 2 to 12 months.^9^, ^10^ Upon diagnosis, current therapies include surgery, radiotherapy, chemotherapy and immunotherapy. However, these therapeutic options fail to improve quality of life and overall survival, as the 3 year survival rate after the development of brain metastases is only 4.8% in treated patients.^7^, ^11^ Subgroups of patients with brain metastases harboring specific molecular alterations can benefit from targeted therapies, such as epidermal growth factor receptor (EGFR), anaplastic lymphoma kinase (ALK), and human EGFR 2 (HER2) inhibitors.^12^, ^13^, ^14^ Unfortunately, these therapies are offered in a limited number of clinical scenarios, therefore strategies to uncover molecular features of brain metastasis are needed to drive development of new targeted therapies.

**FIGURE 1:**
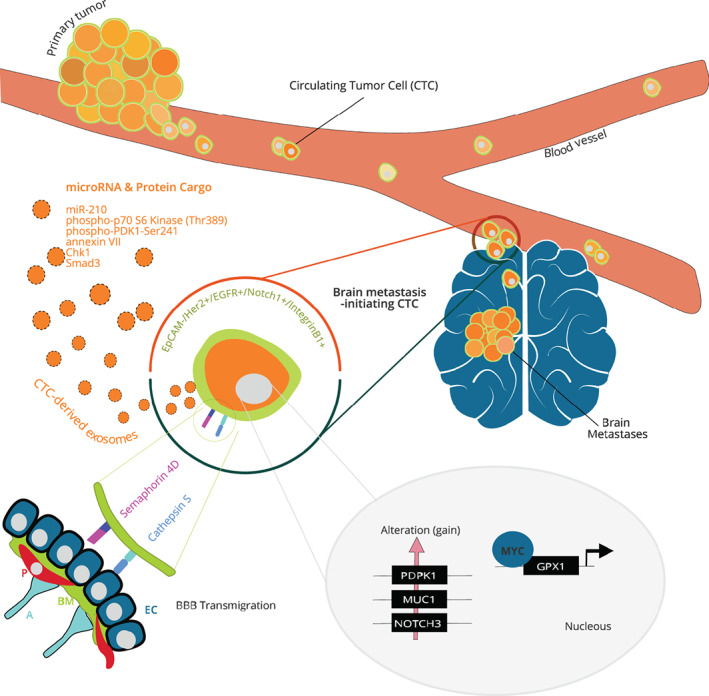
Determinants of brain metastasis‐initiating circulating tumor cells. Tumor cells from the primary tumor may spread to the brain through the blood. CTCs are present in very low concentrations in the blood of cancer patient; however, a small subset of CTCs is expected to be uniquely capable of extravasation thought the BBB. The molecular features of these brain metastasis‐initiating CTCs have been studied, and some of the key molecules involved in the brain tropism are summarized in this figure. At the cell surface, brain‐tropic CTCs are negative for EpCAM and enriched for Her2, EGFR, Notch1, and integrin B1.^16^, ^17^, ^18^ The transmembrane receptor Semaphorin 4D and proteinase Cathepsin S are upregulated in brain‐tropic CTCs and facilitate transmigration of the BBB.^19^, ^20^ Exosomes isolated from brain‐tropic CTC are enriched in miR‐210, phospho‐p70 S6 Kinase (Thr389), annexin VII, phospho‐PDK1‐Ser241, Chk1, and Smad3.^21^ Copy number alterations (gain) have been detected for PDPK1, MUC1, and NOTCH1 genes in brain‐tropic CTCs.^22^ The transcription factor MYC is upregulated in breast cancer brain‐tropic CTCs and promotes the antioxidant enzyme GPX1 expression. MYC/GPX1 mitigate the oxidative stress elicited by activated microglia.^20^ A, astrocyte; BBB, blood‐brain barrier; BM, basement membrane; CTC, circulating tumor cell; EC, endothelial cell; EpCAM, epithelial cell adhesion molecule; P, pericyte

Mechanisms that govern brain metastasis are thought to be complex and involving multiple drivers. Addressing the mechanism of brain metastasis requires an understanding of the dynamic interplay between metastatic cells and the brain microenvironment that is crucial for successful tumor growth. The brain microenvironment is unique due to the tight control imposed by the blood‐brain barrier (BBB) and blood‐cerebrospinal fluid (CSF) barrier to prevent breaches by most immune and tumor cells. This protective barrier is composed by brain endothelial cells with unique features of tight junctions coupled with low transcytosis rate.^15^ In addition, pericytes encapsulated by a basement membrane and the glia limitants of the astrocytes contribute to the BBB functions (Figure 1). The blood‐CSF barrier between choroid plexus blood vessels and the CSF is formed by choroid plexus epithelial cells that are joined via tight junctions.^23^ In the choroid plexus, blood vessels are fenestrated and present intercellular gaps, forming a nonrestrictive barrier. Compromising these barriers of the brain constitutes a crucial first step in the metastatic colonization, and recent studies demonstrated that the interaction of tumor cells with brain endothelial cells is critical for crossing the BBB.^24^, ^25^, ^26^ However, current disease models for brain metastasis are inadequate and most routinely cultured cancer cell lines fail to efficiently metastasize to the brain in animal models.^27^, ^28^ Thus, interest abound in developing new experimental models to improve our limited knowledge of the physiopathology of brain metastasis.

Of the various models used to study brain metastasis, the triple‐negative breast cancer cell line, MDA‐MB‐231, has been widely used to generate a subline with enhanced capacity for forming brain metastasis by in vivo passaging in mice.^24^ Extensive studies using this model provided important information on brain metastasis^24^, ^25^, ^29^, ^30^, ^31^, ^32^ but has the drawback to be limited to one cell line from one cancer subtype. Although other brain‐tropic cell lines or patient‐derived xenografts models have been developed, they only partially recapitulate the complex metastatic process.^7^ Thus, efforts in establishing new models of brain metastasis are needed to identify the underlying molecular mechanisms that allow patient‐derived precursors of brain metastasis to transit to the brain. These precursors belong to a subset of circulating tumor cells (CTCs) that enter the bloodstream and are expected to be uniquely capable of extravasation through the BBB. However, these brain metastasis‐initiating CTCs have yet to be fully characterized due to the challenge of analyzing this unique cell resource.

## 2 APPROACHES FOR PRECLINICAL CTC STUDIES

During tumor progression, tumor cells invade the primary or metastatic tumor microenvironment and intravasate into the circulation, where they are referred as CTCs (Figure 1). Once in the bloodstream, CTCs must survive to disseminate throughout the body. A gradually decreasing proportion of CTCs is capable to arrest within the vasculature, extravasate into specific organs, and proliferate to form tumor at a secondary site.^33^ Brain exposure to CTCs is thought to be high due to the dense microcapillary network and high blood flow of this organ.^1^ However, it is known that most of the arrested CTCs will die, suggesting that cells that go on to form metastases must have adaptive mechanisms that allow them to persist and grow in the local microenvironment.^26^ Molecular features of the small subset of brain metastasis‐initiating CTCs remain incompletely understood, mostly because CTCs are present in very low concentrations in the blood of cancer patient.^34^ Due to the technological advances in enrichment and detection of viable rare cell, it is now feasible to characterize the biological properties of CTCs. Over the past decades, numerous new CTC capture devices have been developed and often combine methods for enrichment and detection. Enrichment strategies can rely on the physical and biological properties of CTCs.^35^ Approaches relying on the physical properties includes size,^36^, ^37^ deformability,^38^ density,^35^ and charge^39^ to discriminate CTCs from blood cells. Common strategies for CTC isolation are based on targeting surface markers that are specifically expressed by the tumor or epithelial cells (positive selection) or the blood cells (negative selection).^40^ These technologies are dependent on specific antibodies that bind to CTC markers (such as epithelial cell adhesion molecule [EpCAM], Her2) or to leukocyte markers (such as CD45 and CD15).

After enrichment, a variety of methods using protein‐based and nucleic‐based approaches have been designed to detect and characterize CTCs from cancer patients' blood.^41^ Among protein‐based methods for detection and characterization of CTCs, flow cytometry,^42^ CTC‐Chip,^43^ RareCyte, and CellSearch platforms^44^, ^45^, ^46^ demonstrated that they can process large sample volume with high specificity. These methods rely on the use of fluorescent‐conjugated antibodies targeting CTC markers such as EpCAM and cytokeratin (CK). The advance in technologies for single cell sequencing have allowed genomic and transcriptomic analysis of individual CTCs from cancer patients. Several studies with successful CTC enrichment followed by epigenetic and transcriptomic analysis at the single cell level revealed molecular features in CTCs involved with cancer metastasis.^47^, ^48^, ^49^ For example, in pancreatic cancer, Ting et al observed that mouse and human CTCs exhibit a high level of stromal‐derived extracellular matrix proteins, which may contribute to pancreatic cancer metastasis.^48^ More recently, Gkountela et al profiled the DNA methylation landscape of single CTCs and CTC clusters. In this study, these authors reported a link between CTC clustering and specific DNA methylation changes that promote stemness and metastasis of breast cancer.^49^

Sufficient studies have proven the prognostic value of CTCs, obtained via a minimally invasive venipuncture procedure, as a real‐time “liquid biopsy” for monitoring active tumor progression and treatment response.^50^, ^51^ The drawback is that CTCs isolated by these methods are fixed and recovered in a low number, limiting the phenotypic characterization of CTCs. To overcome this limitation, different methods have been established to expand CTCs in vitro and in vivo in order to elucidate the functional properties of viable metastasis‐initiating CTCs. Developing in vitro CTC culture has been challenging, mostly because of the scarcity of CTCs recovered from each patient's blood sample.^34^ Despite this challenge, several groups have successfully established CTC lines from cancer patients at advanced stages. Yu et al established six long‐term maintained CTC lines from luminal‐type breast cancer patients and revealed the promises of using CTC lines for drugs sensitivity testing and potential new therapeutic targets identification.^43^ Moreover, Cayrefourcq et al expanded colon cancer CTCs in vitro via long‐term culture.^52^ This CTC line shares similar main features with the primary tumor and presents stem cell‐like characteristics with intermediate epithelial/mesenchymal phenotype. These studies showed that CTC expanded in vitro are tumorigenic in mice, thus representing a promising model for dissecting mechanisms regulating the metastatic cascade. Beside breast and colon cancer, in vitro culture of human CTCs have been reported in prostate and lung cancer.^53^, ^54^ However, there are concerns that CTC lines can be established mainly from metastatic cancer patients exhibiting high CTC counts and that CTCs expanded in vitro may not preserve the cancer molecular heterogeneity. Thus, alternative approaches to expand freshly isolated CTC directly in vivo have been considered. For example, isolated CTCs from patient blood can be directly injected into immunodeficient mice and be expanded in vivo to obtain CTC‐derived xenograft (CDX). Several groups have used this approach with CTCs isolated from different cancer types, including breast, small cell lung cancer (SCLC), and non‐SCLC (NSCLC), to demonstrate the tumor‐initiating properties of CTCs when they were directly injected into femoral bone or flank of immunodeficient mice.^44^, ^55^, ^56^ These recent advancements in the isolation and expansion of patient‐derived CTCs offer significant opportunities to enhance our current knowledge about metastasis. In the rest of this review, we will discuss the recent research advances in the field of CTCs to understand the brain metastasis cascade in solid tumor.

## 3 PROGNOSTIC SIGNIFICANCE OF CTCS IN PATIENTS WITH BRAIN METASTASES

From a clinical point of view, screening CTCs as an early detection tool of cancer progression and monitoring of treatment effectiveness is well documented in various types of cancer.^43^, ^57^, ^58^, ^59^ In the case of brain metastasis, intracranial lesions can develop years after primary tumor removal. Therefore, there is a need for innovative approaches to improve brain tumor risk assessment and treatment evaluation. Screening CTCs could be a promising noninvasive way to monitor brain tumor. Several studies have tried to use CTCs as prognostic biomarker to assess brain metastasis.^60^ While they often conclude that higher CTC levels were strongly associated with worse survival, they failed to show an association between CTC count and brain metastasis.^61^ It has been suggested that brain metastatic (BM) patients exhibit a reduced frequency of CTCs compared with patients with other metastases using EpCAM‐based CTC enumeration.^62^ However, two studies reported that CTCs harboring high competence to generate brain metastasis do not express EpCAM.^16^, ^17^ For established brain tumors, CTCs have also been analyzed in several contexts. In the case of primary brain tumor, isolation of circulating brain tumor cells from patient with glioblastoma (GBM) has been challenging in the past years, mostly because brain tumor cells lack the expression of biomarkers used for most of CTC detection strategies (EpCAM, CD326).^63^, ^64^ Recent alternative CTC isolation approaches were able to demonstrate that GBM patients harbor CTCs in the peripheral blood.^63^, ^64^, ^65^ GBM CTCs were rare and showed invasive mesenchymal characteristics and additional mutations absent in the primary tumor. Similarly to other cancer types, GBM patients with progressive disease harbored higher frequency of CTCs.^64^ The utility of CTC count to evaluate the efficacy of brain metastasis treatment has been investigated in HER2‐positive breast cancer patients. In patients with newly diagnosed brain metastasis, this clinical trial suggested that early clearance of CTCs detected by CellSearch in patients' blood was correlated with brain metastasis response to a targeted therapy in HER2‐positive breast cancers.^66^

## 4 MOLECULAR PROFILING OF CTCS IN PATIENTS WITH BRAIN METASTASES

It has been shown that CTCs are quite heterogeneous. Therefore, molecular profiling of CTCs competent for brain metastasis is needed. One study assessed the molecular features of CTCs and identified a signature of brain metastasis.^16^ Authors of this study isolated and characterized CTCs from 38 breast cancer patients. They identified a brain metastasis CTC signature comprising markers of HER2^+^/EGFR^+^/HPSE (human heparanase)^+^/Notch1^+^ and lacking the EpCAM. Additionally, they generated CTC lines and demonstrated that those expressing the brain metastasis signature were highly invasive and competent to generate brain and lung lesions when xenografted in immune‐compromised mice. Authors suggested that this brain metastasis CTC signature could be used to target brain metastasis‐initiating CTCs. Interesting follow‐up studies from the same team further characterized breast cancer CTCs competent for brain metastasis.^17^, ^67^ Comparison between brain metastasis‐associated CTCs and CTCs from other metastatic sites showed that Notch and immune evasion signaling were enriched in CTCs derived from patients with brain metastasis.^17^ Moreover, CTC subsets were selected for urokinase receptor (uPAR) and integrin β1 positivity, two markers implicated in breast cancer dormancy. These subsets showed proliferative and invasive properties in vitro.^67^ However, additional studies will be needed to assess the predictive value of these CTC subsets for patients at high risk of developing brain metastasis. In a similar approach, Riebensahm et al compared copy number alteration (CNA) profiles of CTCs and corresponding tumor tissue (primary and brain metastases) from three brain metastatic breast cancer (BMBC) patients.^22^ Their study indicated that CNA profiles of CTCs resembled those of primary tumors but most of CTCs within one patient showed a high clonality. Potential brain metastasis‐related aberrations were analyzed, and only one region was gained in all patient‐derived CTCs located in chromosome 1q22‐q23.2 containing, among others, the gene *MUC1*, often used as a diagnostic marker for metastatic progression.^68^ Alterations in pathways known to be involved in brain metastasis were also reported, including notch (NOTCH3 gain) and PI3K (PDPK1 gain). In addition, mutation analysis in BMBC patient‐derived CTCs showed the most frequent mutated genes belonged to cell cycle regulators (TP53, RB1, and CDKN2A), the PI3K pathway (PTEN, PIK3CA) and regulators of the epithelial‐mesenchymal transition (CDH1) and chromatin remodeling (ARID1A). More recently, a DEPArray‐based screening of CTCs from triple‐negative breast cancer (TNBC) patients discovered a subset of HER2 positive CTCs harboring nuclear dual specificity phosphatase 6 (DUSP6).^18^ In brain metastases, DUSP6 is predominantly nuclear, in contrast to the non‐nuclear pattern in primary and lung metastases of TNBC patients. Therefore, this study suggested that nuclear DUSP6 expression in HER2 positive CTCs could be of high risk of brain metastasis in TNBC patients. These studies on molecular characterization of CTCs have provided new insights into brain metastasis that merit further investigations with large cohort studies. Figure 1 and Table 1 summarize key results related to the molecular profile of brain metastasis‐initiating CTCs. Moreover, functional characterizations are needed in order to discover potential druggable targets in brain metastasis‐initiating CTCs.

**TABLE 1:** Molecular profile of brain metastasis‐initiating circulating tumor cells

Molecule	Finding Description	Reference
EpCAM	Absence of EpCAM expression in CTCs derived from BMBC	Zhang et al^16^
HER2	Identified in a subset of CTCs harboring high competence for brain metastasis. CTCs derived from triple‐negative BMBC patient express HER2	Zhang et al^16^ and Wu et al^18^
EGFR	Identified in a subset of CTCs harboring high competence for brain metastasis. CTC derived from triple‐negative BMBC patient express EGFR	Zhang et al^16^
HPSE	Identified in a subset of CTCs harboring high competence for brain metastasis	Zhang et al^16^
Notch1	Identified in a subset of CTCs harboring high competence for brain metastasis. Notch activity is a feature of BMBC CTCs	Zhang et al^16^ and Boral et al^17^
Notch3	Copy number alteration (gain) detected in CTC lines derived from BMBC	Riebensahm et al^22^
uPAR/integrin β1	EpCAM‐negative CTCs expressing uPAR/intB1 were detected in the blood of patients whose breast cancer had metastasized to the brain	Vishnoi et al^67^
MUC1	Copy number alteration (gain 1q22‐q23.2) detected in CTC lines derived from BMBC	Riebensahm et al^22^
PDPK1	Copy number alteration (gain) detected in CTC lines derived from BMBC	Riebensahm et al^22^
TP53, ARID1A, CDH1, TTN	Most frequently mutated genes in CTC lines derived from BMBC	Riebensahm et al^22^
Nuclear DUSP6	DUSP6 protein is predominantly nuclear in HER2‐positive CTCs and brain metastases of TNBC patients	Wu et al^18^
miR‐210	Upregulated miRNA in exosomes from BM vs non‐BM CTC line	Camacho et al^21^
miR‐19a, miR‐29c	Downregulated miRNA in exosomes from BM vs non‐BM CTC line	Camacho et al^21^
phospho‐p70 S6 Kinase‐Thr389, annexin VII, phosphor‐PDK1‐Ser241, Chk1, Smad3	Upregulated proteins in exosomes from BM vs non‐BM CTC line	Camacho et al^21^
ACC1, TFRC, TSC1, Bcl‐x_L_	Downregulated proteins in exosomes from BM vs non‐BM CTC line	Camacho et al^21^
Cathepsin S	CTSS was highly expressed in SCLC CTC lines	Rath et al^19^
Semaphorin 4D	SEMA4D promotes brain metastasis by enabling breast cancer CTC lines to cross the blood–brain barrier	Klotz et al^20^
MYC, GPX1	MYC upregulates the antioxidant enzyme GPX1 expression in breast cancer CTCs. MYC/GPX1 mitigate the oxidative stress elicited by activated microglia	Klotz et al^20^

Abbreviations: BM, brain metastatic; BMBC, brain metastatic breast cancer; CTC, circulating tumor cell; EpCAM, epithelial cell adhesion molecule; SCLC, small cell lung cancer; TNBC, triple‐negative breast cancer.

## 5 DETERMINANTS OF BRAIN METASTASIS‐INITIATING CTCS

To elucidate which factor could be of relevance as they can affect the BM potential of CTCs, the use of viable patient‐derived CTCs expanded in vitro represents a promising approach. Using a CTC line established from a triple negative BMBC patient,^16^ the authors further generated a CTC brain metastasis‐selected markers variant (CTC1BMSM) from the parental CTC line (CTC1) and investigated the differential microRNA and protein cargo of exosomes isolated from these CTC lines.^21^ Increasing evidence suggested that tumor‐derived exosomes have the potential for regulating tumor survival and organ‐specific metastasis.^69^ In BM CTC‐derived exosomes, authors identified one upregulated (miR‐210) and two downregulated miRNAs (miR‐19a and miR‐29c) compared with the parental CTC line. This result was validated in BM (70W and MDA‐MB‐231BR) vs non‐BM (MeWo and MDA‐MB‐231P) tumor cells. Additionally, the differential proteomic content of BM vs non‐BM cells‐derived exosomes was analyzed. In the CTC1BMSM exosomes, five proteins were upregulated (phospho‐p70 S6 Kinase‐Thr389, annexin VII, phosphor‐Ser241, Chk1, and Smad3) and four proteins were downregulated (ACC1, TFRC, TSC1, and Bcl‐x_L_). Although this study was the first to indicate that viable CTCs expanded ex vivo could help profile brain metastasis‐initiating CTCs, there were no functional validations for the identified factors. Viable patient‐derived CTC lines from SCLC patients were also analyzed in a recent report.^19^ Two SCLC CTC lines were used to screen the expression of 35 proteases. In contrast to other cell lines established from local metastases, metalloproteinase‐9 (MMP‐9) was highly expressed in CTC lines. Interestingly, several members of the cathepsin family were highly expressed in CTCs, with cathepsin S exclusively found in these two CTC lines. Although functional validation was not performed in this study, cathepsin S has been previously described for its role in brain‐specific metastasis.^25^ Key recent findings in brain metastasis were derived from luminal‐type breast cancer CTC lines.^20^ One goal of this study was to assess whether CTCs isolated from patients can generate metastases with similar tropism in a mouse model. When CTC lines were directly injected in the bloodstream of immunodeficient NSG (NOD scid gamma) mice, they formed metastatic lesions to the brain, lung, bone, and ovary—common sites for secondary tumors in breast cancer patients—and showed tropisms for the same organs as those diagnosed in the corresponding patients. One CTC line showed a preferential tropism for the brain, and interestingly, the corresponding patient developed brain metastasis 1 year after CTC isolation. This finding suggests that brain metastasis‐initiating CTCs could be identified early, indicating potential predictive value for brain metastasis. Moreover, this study identified markers of brain metastasis‐initiating subpopulation of CTCs—Semaphorin 4D (SEMA4D) and MYC. In breast cancer, CTCs expressing SEMA4D demonstrated a tendency to metastasize to the brain in mice by enabling CTCs to cross the BBB. Once in the brain microenvironment, MYC acts as a cofactor to facilitate CTC adaptation by mitigating the oxidative stress elicited by activated microglia. A potential mechanism of MYC‐driven brain metastasis is dependent on the upregulation of the antioxidant enzyme glutathione peroxidase (GPX1) by MYC‐positive CTCs. High SEMA4D and GPX1 expression at the primary site correlated with significantly decreased brain metastasis‐free survival, further implicating these genes as potential therapeutic targets for preventing brain metastasis in patients. Prospective studies are needed to provide clinical evidence for the value and benefits of SEMA4D and MYC expression in CTCs as early predictive factors for brain metastasis.

## 6 FUTURE DIRECTIONS

Multiple strategies are being pursued to improve our understanding of brain metastasis. As the mechanisms of brain metastasis are complex and differ between cancer types and patients, analyzing tumor cells representing more tumor tissues will be essential for expanding our knowledge on brain metastasis and guiding clinical decision. In this regard, CTCs have the advantage of allowing more frequent and minimally invasive means for studying and monitoring of disease. The emergence of studies deciphering biomarkers of brain metastasis‐initiating CTCs has promise for improving the prevention of brain metastasis. Future clinical trials will be needed to assess clinical utilities of CTC‐derived BM markers for identifying patient with high risk for brain metastasis. Although the idea of stopping CTCs in their track before they disseminate to secondary tissues seem daunting, existing studies have shown promises in the development of CTC‐targeting therapies. For example, evidences support a functional role of CTC cluster in increasing metastatic potential.^49^, ^70^ Thus, the use of drugs to inhibit CTC clustering in preclinical models has shown exciting results and may lead to future trials to evaluate its therapeutic impact. Recent advances in more efficient next‐generation sequencing technologies should greatly improve our ability to uncover molecular pathways in BM disease. Multiomic single‐cell sequencing data of brain‐tropic CTCs will be feasible to obtain, and when compared with matched primary tumor and non‐brain metastases, can provide novel insights into the unique properties of brain tropism. To facilitate this research direction, a multidisciplinary team including primary oncologist, neurosurgeon, biologist, and computational biologist is critical. Furthermore, although our knowledge of the biology of brain macrometastases is increasing, understanding the early events that govern the molecular changes at the premetastatic niche and that sustain dormancy is greatly needed. Futures studies in this direction should compare CTC‐derived experimental models capable for brain macrometastases to micrometastases or dormant models. Insight from CTC biology may also contribute to future therapeutic developments for treating brain metastases. Advances in understanding the brain tumor microenvironment interaction will potentially provide novel therapeutic strategies. Along this line, new interesting mechanisms regulating interactions of tumor cells with endothelial cells, pericytes, astrocytes, microglia, and neurons have been explored.^20^, ^30^, ^31^, ^71^, ^72^ For example, efforts on a better characterization of the blood‐tumor barrier (BTB) permeability may have profound impact on drug efficacy for brain metastases.^73^, ^74^ These discoveries will be critical for the development of new therapies targeting microenvironmental modulation with the ability to prevent or treat established brain metastases. Although all these preclinical evidences have yet to be translated into patients, they provide considerable hope to benefit patients with brain metastases in the future.

## CONFLICT OF INTEREST

M. Yu is the founder and director of CanTraCer Biosciences Inc. R. Klotz declares no competing interests.

## AUTHORS' CONTRIBUTIONS

All authors had full access to the data in the study and take responsibility for the integrity of the data and the accuracy of the data analysis. Conceptualization, R.K., M.Y.; Methodology, R.K., M.Y.; Investigation, R.K., M.Y.; Formal Analysis, R.K., M.Y.; Resources, R.K, M.Y.; Writing ‐ Original Draft, R.K.; Writing ‐ Review and Editing, R.K., M.Y.; Visualization, R.K., M.Y.; Supervision, M.Y.; Funding Acquisition, M.Y.

## Data Availability

Data sharing is not applicable to this article as no new data were created or analyzed in this study.
